# Effect of Age on Childbearing in Port Harcourt, Nigeria

**Published:** 2015-06

**Authors:** Ngozi C. Orazulike, Israel Jeremiah, Kinikanwo I. Green, Samuel A. Uzoigwe

**Affiliations:** 1 Department of Obstetrics and Gynaecology, University of Port Harcourt Teaching Hospital, Port Harcourt, Rivers State, Nigeria;; 2 Department of Obstetrics and Gynaecology, Niger Delta University, Wilberforce Island Bayelsa State, Nigeria

**Keywords:** Pregnancy, Outcomes, Advanced maternal age, Port Harcourt

## Abstract

**OBJECTIVES:**

To determine the effect of maternal age on pregnancy outcomes in women aged 40 years and above at the University of Port Harcourt Teaching Hospital.

**METHODS:**

A retrospective comparative study was conducted on women aged ≥40 years (n=249) and a control group aged 20–29 years (n=249) who delivered at ≥28 weeks gestation between January 1, 2008 and December 31, 2012. The medical records of the patients were analyzed using Epi Info 6.04d. Association between maternal age and selected obstetrical variables were assessed using the chi-squared and the two-tailed Fisher exact test. Differences were considered statistically significant when *p*≤0.05.

**RESULTS:**

The mean age of the women in the study group was 41.2 ±1.75 versus 26.10 ± 2.37 in the control group. Advanced maternal age was associated with a significantly higher rate of hypertensive disorders of pregnancy (*p*=0.01), diabetes mellitus (*p*<0.01), abnormal lies/presentation (*p*=0.04), caesarean deliveries (*p*<0.01) and low birth weight (*p*=0.04).

**CONCLUSION:**

Older parturients have a higher risk of medical disorders of pregnancy. They are more likely to deliver by caesarean section and have low birth weight babies than their younger counterparts.

## INTRODUCTION

Advanced maternal age defined as maternal age 40 years or greater at the time of delivery is associated with an increased incidence of adverse pregnancy outcomes including aneuploidy and multiple gestations (1, 2). That notwithstanding, the number of older women giving birth has increased worldwide. In South Australia, for example it has increased from 4.6% in 1981 to 21.1% in 2009 and in the United States of America, older women comprise the only age group whose birth rate is on the rise increasing by 6% for those age 40-44 years between 2007 and 2009 (3, 4).

Various reasons have been suggested for this increase including delay in marriage, more focus on education and careers, effective birth control, fertility problems and advances in assisted reproductive technology (ART) (5, 6). There is a natural age related decline in fertility in older women however, with ART, successful pregnancies are not uncommon even in postmenopausal women (7). In less resourced countries, contrary to what obtains in more resourced countries, childbearing is commoner in advanced age multipara due to lack of or ineffective family planning methods, favourable cultural disposition towards large family size and poverty (8).

There are several publications on pregnancies in women aged 40 years or older with conflicting outcomes. Some authors for example have reported an association with preterm delivery, low birth weight, perinatal mortality and higher rate of caesarean section while others have reported no obvious difference in the perinatal outcomes, obstetric outcomes, birth weight, Apgar scores and admission to the neonatal intensive care unit (9-12). No previous studies have been done in our setting on this subject.

This aim of this study was therefore, to determine the impact of maternal age on pregnancy outcome among women delivering at the University of Port Harcourt Teaching Hospital.

## MATERIALS AND METHODS

This was a retrospective comparative study involving 249 of women aged ≥40 years and 249 women aged 20–29 years who served as controls that delivered at ≥28 weeks gestation between January 2008 and December 2012 at the Department of Obstetrics and Gynaecology of the University of Port Harcourt Teaching Hospital (UPTH), Port Harcourt, Rivers State.

Two hundred and forty nine consecutively booked women aged 40 years and above regardless of their parity at gestational age ≥28 weeks were included in the study group and their pregnancy outcome was compared with that of 249 women aged 20-29 years selected randomly who served as controls. Unbooked women, women with multiple pregnancies, women less than 20 years and women who had pre-existing medical conditions such as hypertension or diabetes mellitus were excluded from the study.

Ethical approval was obtained from the ethical review committee of the University of Port Harcourt Teaching Hospital.

The data were collected from the labour ward delivery register, theatre records and the patient’s case notes obtained from the main records department.

Information extracted from the records included demographics, parity, gestational age, complications including hypertension in pregnancy, gestational diabetes, antepartum haemorrhage, gestational age at delivery, type of delivery, pregnancy outcome including birth weight, perinatal mortality, Apgar scores and Neonatal Intensive Care Unit (NICU) admission.

Data analysis was performed with Epi info ver 6.04d. Association between maternal age and selected obstetric and neonatal variables were assessed using the chi-square and the two-tailed Fisher exact test. Differences were considered statistically significant when *p*≤0.05. The results are presented as mean with standard deviations, percentages, rates and proportions.

## RESULTS

Over the five-year period, 249 (1.75%) out of 14,200 deliveries were in women aged ≥40 years. The mean age of women in the study group was 41.2 ± 1.8 years and that in the control group was 26.1 ± 2.4 years. The older mothers had a higher median parity (2) compared with the control group [0]. Of the older women, 4% [10] had a history of subfertility and required in vitro fertilization to get pregnant compared with 1(0.4%). The difference was statistically significant (*p*=0.01).

Table 1 shows the association between maternal age and obstetric outcome. Maternal age was significantly associated with hypertensive disorders of pregnancy, 11.2% in the study group compared to 4.0% in the control group (*p*=0.01). The incidence of gestational diabetes mellitus was higher in the advanced maternal age group (5%) compared to the control group (0%) (*p*<0.01) and likewise abnormal lie and presentation (4.4% vs 1.2%) (*p*=0.04). Uterine fibroids complicating pregnancy were significantly commoner among the study group (4.8%) than the control group (0.8%) (*p*=0.01). Antepartum haemorrhage, postpartum haemorrhage, urinary tract infection and genital tract trauma were commoner in older mothers but the differences were not statistically significant. Caesarean delivery was more common in the advanced maternal age group (60.9%) compared to the control group (17.9%) (*p*<0.01). The infants of the older women were significantly likely to be low birth weight (12.1% versus 6.3%) (*p*=0.04). The incidence of low Apgar scores in the fifth minute among babies born to the older mothers was higher (7.8%) than for those born to the younger women (4.6%). The difference was however not statistically significant. Although congenital malformations were higher in the advanced maternal age group, the difference was not statistically significant. The perinatal mortality was higher among the study group as 3 intrauterine foetal deaths occurred in this group while no perinatal deaths were recorded among the controls.

**Table 1:** Relationship between maternal age and pregnancy complications

	Complications	Study group Freq. (%)	Control group Freq. (%)	Chi square x^2^	*P* Value

1.	Hypertensive disorders of pregnancy	28 (11.2)	10 (4.0)	7.01	0.01
2.	Gestational diabetes mellitus	5 (2.0)	0 (0)		<0.01^a^
3.	UTI in pregnancy	3 (1.2)	1 (0.4)		0.32^a^
4.	Uterine fibroids complicating pregnancy	12 (4.8)	2 (0.8)	5.95	0.01
5.	Antepartum Haemorrhage	9 (3.6)	3 (1.2)	2.02	0.16
6.	Abnormal lies and presentation	11 (4.4)	3 (1.2)	4.44	0.04
7.	Postpartum haemorrhage	11(4.4)	5 (2.0)	1.49	0.22

a Fisher exact *p*-value.

## DISCUSSION

Our results on 249 mothers of advanced age have shown a significantly increased incidence of hypertensive disorders in pregnancy, gestational diabetes mellitus, abnormal lies and presentation and caesarean deliveries in the mothers of advanced age as reported in previous studies (1, 2, 13-16). The prevalence of diabetes and hypertension are known to increase with age and related to vascular endothelial damage that occurs with aging (17). Pancreatic β-cell function and insulin sensitivity fall with age.

The rates of antepartum haemorrhage, postpartum haemorrhage, genital tract trauma, were higher in the older women, though not statistically significant. This could be explained by the increased incidence of malposition especially breech and increased incidence of uterine fibroids complicating pregnancy.

In our study, women aged 40 years and above were at a higher risk for caesarean delivery when compared to younger women aged 20–29 years (60.9% vs 17.9%), a finding in keeping with previously published studies (2, 12, 18, 19). It is thought that aging leads to inefficient uterine action from decreased myometrial efficiency and dystocia in labour. Additionally in older primigravid mothers, partly due to previous infertility and low fecundity, both patients and obstetricians adopt a more active approach of elective caesarean delivery, thereby increasing the caesarean section rate.

Uterine fibroids complicating pregnancy was significantly higher among the older women in our study. They were associated with pregnancy complications such as preterm birth and lower birth weight. They also contributed to the increased caesarean section rate.

The advanced maternal age mothers had a significantly higher rate of low birth weight babies in our study as corroborated by others (13, 15, 20). The incidence of congenital malformation was higher among the older women, though the difference was not statistically significant. This is similar to other studies (21). The risks of aneuploidy and fetal congenital anomalies increases with maternal age thus perinatal death (PND) seems to have a higher prevalence among newborns of older women, as reported by Franz *et al* (22, 23). The PND may be due perhaps to deficiency of placental perfusion caused by poor uterine vascularization. Also there is an association between maternal age and certain risk factors for fetal death e.g. chronic diseases and obstetric complications. This was demonstrated in our study as the 3 stillbirths occurred in the older age group. There is some controversy regarding the perinatal outcome in older pregnant women. Olusanya *et al* in a study of 513 advanced maternal age mothers in a tertiary hospital in Nigeria reported no significant adverse perinatal outcome in advanced maternal age women compared to younger mothers (24). They concluded that advanced maternal age was not necessarily associated with adverse perinatal outcomes in settings with good antenatal follow-up and favourable maternal disposition to planned caesarean section.

Our study was retrospective so there were some missing data. It did not include some other factors known to influence pregnancy outcome such as infectious diseases and socio-economic status of the mothers (educational level and employment status). Thus, further research especially in the form of prospective studies in a large cohort of women is necessary especially for our environment.

In conclusion women of advanced maternal age (regardless of their parity) have a higher risk of diabetes mellitus and hypertensive disorders of pregnancy. They are at increased risk of caesarean delivery and low birth weight babies than their younger counterparts. These adverse outcomes should not, however, contraindicate pregnancy. They should be counseled adequately so they are well informed before conception.
